# Plasma‐derived Extracellular Vesicle Surface Antigens related to Platelets as biomarker for Alzheimer’s Disease

**DOI:** 10.1002/alz.083919

**Published:** 2025-01-09

**Authors:** Elena Vacchi, Carlotta Ginevra Cimiotti, Sara Bolis, Ankush Yadav, Emanuele Pravatà, Sandra Pinton, Stefania Rossi, Paolo Paganetti, Alain Kaelin‐Lang, Lucio Barile, Leonardo Sacco, Giorgia Melli

**Affiliations:** ^1^ LRT‐EOC, Bellinzona, Ticino Switzerland; ^2^ Neurocenter of Southern Switzerland, Lugano, Ticino Switzerland

## Abstract

**Background:**

Alzheimer’s disease (AD) is the most common irreversible neurodegenerative disorder of the elderly. Its clinical diagnosis can be confirmed with expensive and invasive laboratory exams. Thus, new biomarkers and diagnostic tools to facilitate the proper management of patients are required. With the aim to identify new biomarkers for AD, we characterized the expression of multiple plasmatic Extracellular Vesicles (EVs) surface markers, related to immune response and inflammation. Indeed, emerging evidence suggests a central role of inflammation in the development and pathogenesis of AD.

**Methods:**

Thirty confirmed AD patients, among which 7 with mild cognitive impairment (MCI) and 23 with moderate‐severe dementia, and 20 healthy controls (HC) were enrolled. Patients underwent clinical evaluation, cerebrospinal fluid Aβ and Tau analysis, and magnetic resonance imaging (MRI). All subjects underwent blood collection. Plasma‐derived EVs were analyzed by flow cytometry to measure the expression of 37 EV surface markers.

**Results:**

AD‐derived EVs showed higher CD62P and lower CD2 expression compared to those from HC. CD62P was associated with AD diagnosis, displaying good diagnostic performance, correlating with cognitive impairment, and showing higher expression in AD patients with pathological levels of Aβ42 in the CSF. These results were re‐confirmed considering the moderate‐severe patients only. In addition, CD41b was found increased in MCI subjects compared to both HC and severe AD showing reliable diagnostic performance for the MCI group.

**Conclusions:**

Plasmatic EV surface markers characterization reinforces the role of EVs as promising biomarkers for AD, opening the possibility of developing non‐invasive diagnostic tools.

